# Bipolar Disorder and the TCI: Higher Self-Transcendence in Bipolar Disorder Compared to Major Depression

**DOI:** 10.1155/2011/529638

**Published:** 2011-07-18

**Authors:** James A. Harley, J. Elisabeth Wells, Christopher M. A. Frampton, Peter R. Joyce

**Affiliations:** ^1^Department of Psychological Medicine, University of Otago Christchurch, P.O. Box 4345, Christchurch Mail Centre, Christchurch 8140, New Zealand; ^2^Department of Pathology, University of Otago Christchurch, Christchurch 8140, New Zealand; ^3^Department of Public Health and General Practice, University of Otago, Christchurch, Christchurch 8140, New Zealand

## Abstract

Personality traits are potential endophenotypes for genetic studies of psychiatric disorders. One personality theory which demonstrates strong heritability is Cloninger's psychobiological model measured using the temperament and character inventory (TCI). 277 individuals who completed the TCI questionnaire as part of the South Island Bipolar Study were also interviewed to assess for lifetime psychiatric diagnoses. Four groups were compared, bipolar disorder (BP), type 1 and 2, MDD (major depressive disorder), and nonaffected relatives of a proband with BP. With correction for mood state, total harm avoidance (HA) was higher than unaffected in both MDD and BP groups, but the mood disorder groups did not differ from each other. However, BP1 individuals had higher self-transcendence (ST) than those with MDD and unaffected relatives. HA may reflect a trait marker of mood disorders whereas high ST may be specific to BP. As ST is heritable, genes that affect ST may be of relevance for vulnerability to BP.

## 1. Introduction

An individual's personality develops early, is stable, and has a strong heritable component. Personality traits have been implicated as factors which influence the predisposition to bipolar disorder and may help to distinguish between major depressive disorder (MDD), bipolar disorder (BP1) and bipolar disorder with hypomania, bipolar type 2 (BP2). Personality traits are also being considered as potential useful endophenotypes for the investigation of the genetic basis of complex mental disorders [1]. The adequate description of personality in bipolar disorder is required to identify profiles and traits that may be useful to enhance the understanding of bipolar disorder. 

The Temperament and character inventory revised (TCI-R) is a 240-item 5-point Likert scale self-administered questionnaire which assesses personality following Cloninger's psychobiological model [2]. This model assesses personality in seven dimensions, four temperament scales: novelty seeking (NS), harm avoidance (HA), reward dependence (RD) and persistence (PS), and three character scales: self-directedness (SD), cooperativeness (CO), and self-transcendence (ST). All of the scales reflect total scores of either four or five subscales (Table 1).

In this study, we applied the TCI-R to a sample of bipolar disorder patients and their relatives with the objective of highlighting differences between diagnostic groups for future investigation. As self-reported personality traits may be affected by current mood state, we were also interested in personality differences between diagnostic groups when the effect of current self-reported mood was taken into consideration.

## 2. Methods

Bipolar probands and their family members were recruited in Christchurch, New Zealand as part of the South Island Bipolar Study [3–5] and interviewed using the Diagnostic Interview for Genetic Studies (DIGS) [6, 7]. All participants also completed the revised 240-item temperament and character inventory (TCI-R) self-report questionnaire [8]. Probands and relatives were not required to be euthymic when completing the TCI, and the beck depression inventory (BDI) [9] was used to assess mood state at the time of questionnaire completion.

Two hundred and seventy-seven individuals comprising 65 bipolar probands (50 BP1 and 15 BP2), 134 first-degree relatives, 70 other blood relatives and 8 spouses were assessed using the DIGS, which resulted in four diagnostic categories, BP1 (*n* = 60, 55% female), BP2 (*n* = 33, 52% female), MDD (*n* = 97, 69% female) and unaffected relatives (*n* = 87, 49% female). Individuals with diagnoses of bipolar disorder and depression not otherwise specified were excluded from analyses. Groups were compared using one-way analysis of variance (ANOVA) with Tukey post hoc analysis. TCI scales significantly different in the ANOVA analyses were corrected for current mood state by including BDI score as a covariate in a univariate analysis of covariance (ANCOVA). As the participants of this study are related, the influence of family clustering was corrected for by including family identification number during sample preparation as a cluster using complex sample preparation with replacement sampling. As this study was explorative, no correction for multiple testing was applied. All statistical analysis was conducted using SPSS version 13 (SPSS Inc., Chicago, Ill, USA). With *α* = 0.05 and power of 80% and ignoring the possible design effect from clustering, the detectable alternative for comparison of the two smallest groups (33 and 65) was 0.6 sd between the means whereas for comparison of the two largest groups (87 and 95) it was 0.4 sd [10].

## 3. Results

The mean values and standard deviations of the TCI scales of the four diagnostic groups are presented in Table 1. Of the seven scales, harm avoidance, self-directedness and self-transcendence were significantly different between diagnostic groups. Post hoc analyses revealed that BP1, BP2 and MDD had higher harm avoidance. Both bipolar groups had lower self-directedness than the unaffected relatives and BP1 had higher self-transcendence than MDD or unaffected relatives.

When the subscales were dissected, significant differences were observed for: HA1 (anticipatory worry versus uninhibited optimism), HA2 (fear of uncertainty), HA4 (fatigability and asthenia versus vigour), SD1 (responsibility versus blaming), SD2 (purposefulness versus lack-of-goal directedness), SD3 (resourcefulness), SD4 (self-acceptance versus self-striving), SD5 (enlightened second nature), ST1 (self-forgetfulness versus self-conscious experience), ST3 (spiritual acceptance versus rational materialism), and ST5 (idealistic versus practical). The Tukey post hoc analyses of these subscales are presented in Table 1. 

In addition to differing in personality, the four groups demonstrated significant differences in depressive mood state at the time of assessment. The total BDI scores for the three mood disorder groups were all higher than the unaffected relatives, and the BP1 group scored higher than MDD group. As differences in personality could potentially be due to current mood-state [11, 12], an ANCOVA was conducted for each of the TCI items which displayed a significant difference between the groups. The TCI scale was the dependent variable and mood diagnosis was the fixed variable. Total BDI score was included in the model as a covariate. Results of these analyses are presented in Table 2.

After correction for self-reported depression, all the mood disorder groups had higher HA than the unaffected relatives. In contrast, only BP1 subjects had higher ST than MDD and controls. Among the subscales, three HA subscales, two ST subscales, and SD3 remained different between diagnostic groups.

## 4. Discussion

In this investigation of personality in a sample of individuals with bipolar disorder and their relatives we have shown differences in personality profiles between diagnostic groups. All groups with mood disorder had higher HA than unaffected relatives. ST was higher only in those with bipolar disorder. Initially SD appeared to be different between bipolar disorder and other groups, but correction for current mood state rendered the difference insignificant. 

High HA has often been reported in bipolar disorder [13–20]. Our analysis shows both BP and MDD to be more harm avoidant than nonaffected relatives, further establishing high HA as a characteristic of susceptibility to mood disorder, and not unique to either MDD or BP. High HA or high neuroticism has consistently been linked to depression; however, this vulnerability likely includes bipolar as well as unipolar depression. 

Differences on other scales found by other authors have not been replicated. Janowsky et al. reported greater NS in bipolar patients compared with patients with unipolar depression [15]. In our investigation, a trend to higher novelty seeking in the BP2 group was observed, but no significant difference was found. Using an earlier version of the TCI with nonexpanded persistence dimension, Osher et al. reported lower persistence in bipolar disorder [14, 21]. In our study three persistence subscales were lower in bipolar, however correction for BDI eliminated all but PS4 (perfectionist versus pragmatist), where the BP2 group was greater than the MDD group but no different from BP1 or the nonaffected relatives. Our results do not support the suggestion that low persistence is a temperamental marker of bipolarity. 

The ST dimension of personality is associated with spirituality and a high score is considered to be an adaptive personality trait, when combined with high SD and CO. When high ST is not found with high SD and CO, Cloninger suggests that a schizotypal personality type emerges [8]. Our findings of higher ST in the BP1 group compared with unaffected relatives or those with a lifetime diagnosis of MDD suggests that BP1 patients experience more otherworldly experience. Higher ST scores in bipolar patients have been reported elsewhere compared with unaffected controls [18, 20] and MDD [17]. It appears that the high HA predisposes towards mood disorder and high ST, which is associated with schizotypy [22], contributes a second hit towards the expression of a bipolar phenotype. The ST scores of the BP2 group were not statistically different from the other diagnostic groups but were intermediate. Given the small numbers of BP2 subjects, further research on ST in BP2 is indicated. 

High ST has been associated with polymorphisms in three genes, vesicular monoamine transporter 2 (VMAT2), [23], the serotonin receptor 1A (HTR1A) [24], and glycogen synthase kinase 3beta (GSK3B). Recently, two risk haplotypes within the VMAT2 gene have been identified for bipolar disorder and schizophrenia in a Spanish cohort [25]. Possession of the T-A-G haplotype at markers rs363420-rs363343-rs363272 conferred a relative risk of 4.4, and the C-C-A conferred a relative risk of 1.8 for bipolar disorder versus controls. A polymophism (-50T/C) in the promoter of glycogen synthase kinase 3*β* (GSK3B), associated with age of onset and lithium response in an Italian bipolar cohort, is associated with the ST subscale ST2 (transpersonal identification). Participants homozygous for the rare C allele were significantly higher than those with the TT and CT genotypes [26].

Differences in serotonin receptor 1A density measured by positron emission tomography (PET) have also been associated with ST, especially the subscale spiritual acceptance [27]. Another PET study using the VMAT2 specific ligand (+)-*α*-[11C] dihydrotetrabenzine found brain stem binding to be higher in bipolar disorder and schizophrenic patients compared to controls. Greater VMAT2 binding was observed in the thalamus of the bipolar patients compared with both schizophrenic and control groups [28]. Future investigation into the mechanisms of these two genes and their associations with bipolar disorder and self-transcendence is warranted. 

High harm avoidance and neuroticism personality traits have been well established in unipolar depression. This analysis suggests that high harm avoidance increases disposition to mood disorder, with other modifying factors, genetic and otherwise, may allow the bipolar phenotype to be expressed. This data could be seen as consistent with the findings of McGuffin et al. who demonstrated the independent inheritance of depression and mania in a twin study [28]. The independence may arise from risk genes for high harm avoidance predisposing to mood disorders in general interacting with risk genes for high self-transcendence to increasing vulnerability to a bipolar mood disorder phenotype.

This is one of the larger studies to have used the TCI to compare the personality traits of individuals with bipolar disorder, with major depression, and with no mood disorder. Larger sample size increases the power of detecting true differences. Furthermore the inclusion of a comparison between BP and MDD allows the dissection of the relevance of personality to bipolarity and to mood disorders in general.

The correction of our data for current mood state is important [12]. The inclusion of BDI in the analysis enables us to infer with more certainty that the differences of personality are important in the context of the diagnostic grouping rather than how differences in current level of depression influence personality. The TCI-R is the most current and complete version available, including persistence subscales and two additional ST subscales.

In this investigation of the personality of bipolar disorder probands and their relatives, we have identified differences in the personality dimensions of HA and ST. High HA reflecting tendency to mood disorder and high self-transcendence appears to be specific to bipolar disorder. For future studies of the genetics of bipolar disorder, high ST may be of interest as an endophenotype, and equally genes that influence ST could be considered candidate genes for bipolarity.

## Figures and Tables

**Table 1:** TCI scales by compared diagnostic group.

	Bipolar I (*N* = 60)		Bipolar II (*N* = 33)		Major depressive disorder (*N* = 97)		Unaffected relatives (*N* = 87)		*F*-statistic	*P* value	Post hoc analysis (Tukey)

	Mean	SD	Mean	SD	Mean	SD	Mean	SD			
Novelty-seeking	18.78	6.66	20.30	6.09	17.98	8.47	17.37	5.73	2.28	.085	
Harm-avoidance	18.78	8.57	17.12	8.52	15.98	6.84	11.91	6.38	11.79	<.001	BP 1, BP 2, MDD > unaffected
Reward-dependence	23.37	6.40	22.61	5.49	23.30	7.13	22.43	5.74	0.42	.742	
Persistence	22.75	10.50	26.58	11.04	24.37	13.34	26.06	8.65	1.84	.146	
Self-directedness	31.13	8.51	30.52	10.91	35.08	10.82	37.34	6.11	10.26	<.001	MDD, unaffected > BP 1, BP 2
Cooperativeness	33.88	6.89	34.33	6.36	35.87	4.22	35.57	5.92	1.94	.131	
Self-transcendence	22.43	12.30	18.79	9.45	17.54	11.15	15.39	10.99	4.04	.01	BP 1 > MDD, unaffected

*Subscales*											
HA1 (anticipatory worry versus uninhibited optimism)	5.00	2.90	5.18	3.08	4.23	2.38	3.03	2.30	8.87	<.001	BP 1, BP 2, MDD > unaffected
HA1 (fear of uncertainty versus confidence)	4.73	1.64	3.82	1.98	4.26	1.82	3.55	1.79	6.25	.001	BP 1, MDD > unaffected
HA3 (shyness with strangers versus gregariousness)	4.13	2.75	3.97	2.86	3.97	2.61	3.14	2.25	2.43	.071	
HA4 (fatigability and asthenia versus vigour)	4.92	3.02	4.15	2.94	3.53	2.32	2.18	1.94	17.94	<.001	BP 1, BP 2, MDD > unaffected BP 1 > MDD
SD1 (responsibility versus blaming)	5.92	2.16	5.55	2.66	6.65	1.66	6.93	1.54	4.99	.003	unaffected > BP 1, BP 2; MDD > BP 1
SD2 (purposefulness versus lack-of-goal direction)	5.25	2.11	5.15	2.09	6.05	1.81	6.30	1.59	5.45	.002	Unaffected > BP 1, BP 2; MDD > BP 1
SD3 (resourcefulness)	3.10	1.70	3.45	1.54	3.86	1.22	4.43	0.94	14.05	<.001	Unaffected > BP 1, BP 2, MDD; MDD > BP 1
SD4 (self-acceptance versus self-striving)	7.97	2.65	7.48	3.28	8.62	2.47	9.06	2.13	3.78	.014	Unaffected > BP 1, BP 2
SD5 (congruent second nature)	8.90	2.92	8.88	3.30	9.91	1.93	10.63	1.76	7.59	<.001	Unaffected > BP 1, BP 2
ST1 (self-forgetfulness versus self-conscious experience)	4.05	2.46	3.88	2.62	2.96	2.38	2.11	1.72	10.63	<.001	BP 1 > MDD, unaffected; BP 2 > unaffected
ST2 (transpersonal identification versus self-isolation)	2.38	2.11	1.79	2.09	1.73	1.81	1.54	1.59	1.82	.15	
ST3 (spiritual acceptance versus rational materialism)	5.87	3.57	5.06	2.83	4.49	3.30	4.28	2.82	3.31	.024	BP 1 > MDD, unaffected
ST4 (enlightened versus Objective)	4.95	4.07	4.06	3.47	4.23	4.37	3.56	4.15	1.29	.285	
ST5 (idealistic versus practical)	5.18	2.45	4.00	2.44	4.12	2.62	3.90	2.81	2.84	.043	BP 1 > MDD, unaffected

Beck depression inventory	10.90	9.83	9.79	9.48	6.91	8.64	2.99	3.68	13.82	<.001	BP 1 > MDD, unaffected; BP 2, MDD > unaffected

**Table 2:** Adjusted means of TCI scales and subscales found to be significant in original ANOVA corrected for mood at time of completing TCI questionnaire using total BDI score as a covariate.

	Bipolar I (*N* = 60)		Bipolar II (*N* = 33)		Major depressive disorder (*N* = 97)		Unaffected relatives (*N* = 87)		*F*-statistic	*P* value

	Adjusted mean	SE	Adjusted mean	SE	Adjusted mean	SE	Adjusted mean	SE		
*Harm Avoidance*	16.99	0.88	15.82	1.23	15.97	0.64	13.65	0.63	3.52	.019
HA1 (anticipatory worry versus uninhibited optimism)	4.44	0.31	4.77	0.41	4.22	0.24	3.58	0.23	2.76	.047
HA2 (fear of uncertainty versus confidence)	4.49	0.20	3.64	0.33	4.26	0.18	3.79	0.19	3.05	.033
HA4 (fatigability and asthenia versus vigour)	4.33	0.34	3.73	0.46	3.52	0.20	2.75	0.2	5.89	.001

*Self Directedness*	33.47	0.88	32.21	1.37	35.10	0.54	35.07	0.68	1.57	.2
SD1 (responsibility versus blaming)	6.47	0.21	5.95	0.33	6.65	0.13	6.39	0.18	1.89	.14
SD2 (purposefulness versus lack-of-goal direction)	5.74	0.24	5.50	0.28	6.05	0.17	5.83	0.17	0.97	.41
SD3 (resourcefulness)	3.43	0.20	3.69	0.20	3.86	0.11	4.10	0.1	3.26	.026
SD4 (self-acceptance versus self-striving)	8.33	0.34	7.75	0.53	8.62	0.25	8.70	0.25	0.9	.44
SD5 (congruent second nature)	9.50	0.33	9.31	0.42	9.91	0.16	10.05	0.21	1.05	.37

*Self Transcendence*	22.13	1.55	18.57	1.69	17.53	1.13	15.69	1.24	3.12	.03
ST1 (self-forgetfulness versus self-conscious experience)	3.82	0.30	3.72	0.45	2.96	0.23	2.33	0.2	5.77	.001
ST3 (spiritual acceptance versus rational materialism)	5.84	0.45	5.04	0.51	4.49	0.34	4.30	0.32	2.93	.038
ST5 (idealistic versus practical)	5.08	0.32	3.92	0.43	4.12	0.26	4.00	0.3	2.26	.088
